# Case Report: Resolution of remitting seronegative symmetrical synovitis with pitting edema during nivolumab therapy for gastric cancer

**DOI:** 10.3389/fonc.2023.1260818

**Published:** 2023-10-05

**Authors:** Hirofumi Ohmura, Moe Kondo, Masato Uenomachi, Hiroshi Ariyama, Mamoru Ito, Kenji Tsuchihashi, Masahiro Ayano, Hiroaki Niiro, Koichi Akashi, Eishi Baba

**Affiliations:** ^1^ Department of Oncology and Social Medicine, Kyushu University Graduate School of Medical Sciences, Fukuoka, Japan; ^2^ Department of Medicine and Biosystemic Science, Kyushu University Graduate School of Medical Sciences, Fukuoka, Japan; ^3^ Department of Diabetes Mellitus and Endocrinology, Nanpuh Hospital, Kagoshima, Japan; ^4^ Department of Oncology, Kitakyushu Municipal Medical Center, Fukuoka, Japan; ^5^ Department of Medical Education, Kyushu University Faculty of Medical Sciences, Fukuoka, Japan

**Keywords:** gastric cancer, RS3PE, irAE, nivolumab, pathology

## Abstract

The anti-programmed cell death-1 (PD-1) antibody nivolumab has been shown to significantly prolong the survival of patients with unresectable advanced or recurrent gastric cancer (AGC). However, immune-related adverse events (irAEs), which show different profiles from those of cytotoxic agents or conventional molecular-targeted drugs including tyrosine kinase inhibitors, have been reported. Remitting seronegative symmetrical synovitis with pitting edema (RS3PE) is a rare autoimmune disorder with acute-onset, rheumatoid factor-negative, symmetric synovitis associated with limb edema observed in elderly persons. A case of RS3PE syndrome that developed after administration of nivolumab for advanced gastric cancer is reported. This is the first report of a case of RS3PE syndrome as an irAE caused by nivolumab in a patient with gastric cancer.

## 1 Introduction

For patients with advanced or recurrent gastric cancer, systemic chemotherapy is the standard therapy. Platinum, fluoropyrimidine, and taxanes are used as cytotoxic agents. Ramucirumab, an angiogenesis inhibitor, is used for human epidermal growth factor receptor 2(HER2)-positive or negative advanced or recurrent gastric cancer (AGC), and trastuzumab and trastuzumab deruxtecan are used for HER2-positive AGC as molecular-targeted agents (1–5). In recent years, the efficacy of immune checkpoint inhibitors against AGC, which is resistant to these agents, has been shown. Nivolumab, a fully human anti-programmed death-1 (PD-1) monoclonal antibody, demonstrated a significant survival benefit for previously untreated AGC patients in combination with chemotherapy and for AGC patients previously treated with two or more chemotherapy regimens (6–8). It is considered that the administration of anti-PD-1 antibody activates tumor-specific T cells in cancer-bearing patients, resulting in an antitumor effect. On the other hand, it is also known that immune-related adverse events (irAEs) based on the autoimmune response, including skin disorders, colitis, and thyroid dysfunction, develop due to non-tumor-specific T cell activation (9), and attention to irAEs is needed.

Remitting seronegative symmetrical synovitis with pitting edema (RS3PE), a disease reported by McCarty et al., occurs more frequently to men over the age of 60 years (male to female ratio, 3:2), and patients develop acute or subacute synovitis accompanied by symmetric pitting edema of the limbs or feet. RS3PE is negative for serum rheumatoid factor and joint destruction on X-ray images and has a good prognosis. RS3PE has a similar pathology to polymyalgia rheumatica (PMR), except that the predominant manifestation of PMR is arthritis. In the treatment of RS3PE, administration of steroids is effective. It has also been reported that 16-30% of patients with RS3PE are associated with malignant tumors such as prostate cancer, colon cancer, gastric cancer, and hematopoietic tumors (10). RS3PE patients with malignant tumors are often resistant to treatment, but some cases showed improvement of symptoms after tumor resection (11). Based on these characteristics, RS3PE is considered one of the paraneoplastic syndromes. However, the mechanism of its onset has still not been elucidated.

RS3PE that developed after administration of nivolumab has been reported in three cases of malignant melanoma and one case of non-small cell lung cancer (12–15). However, there has been no report of a gastric cancer case that developed RS3PE after the administration of nivolumab. In addition, histological evaluation of an RS3PE case after administration of nivolumab has not been performed. The first gastric cancer case that developed RS3PE after the administration of nivolumab is presented.

## 2 Case description

A 73-year-old man developed black stool in January 2017 and consulted a family doctor. Upper gastrointestinal endoscopy showed type 3 advanced gastric cancer (pathological diagnosis: moderately to poorly differentiated adenocarcinoma, HER2-negative) with main lesions from the body to the antrum of the stomach. He had hypertension, aortic stenosis, type II diabetes mellitus, and dyslipidemia since the age of about 50 and he was treated by a family doctor. There was no family history of malignant tumor or collagen disease. He smoked about one pack of cigarettes a day for 48 years. He was referred to our hospital for treatment. His general condition was Eastern Cooperative Oncology Group performance status (ECOG PS) 1, and there were no abnormal physical findings other than pallor of the palpebral conjunctiva.

On close examination, the primary lesion and regional lymph node metastasis were found, and the clinical stage was diagnosed as cT4N3aM0, cStage IIIB. Though laparoscopic curative surgery was planned, peritoneal dissemination was observed during the operation, and he was diagnosed with unresectable gastric cancer (sT4a (SE) N2P1CY0H0, sStage IV). From February 2017, S-1 + oxaliplatin therapy (S-1: 120 mg/day, day 1-14, oxaliplatin: 100 mg/m^2^, day 1, 3-week cycle) was administered.

In April, a tonic-clonic seizure occurred after the second administration of S-1+oxaliplatin. No abnormalities were found on head magnetic resonance imaging, but it could not be ruled out that S-1 was the cause, and chemotherapy was switched to second-line therapy with paclitaxel. An increase in size of abdominal lymph nodes was observed on computed tomography (CT), and progressive disease (PD) was confirmed by response evaluation criteria in solid tumors (RECIST) criteria version 1.1 (16).

From November, nivolumab monotherapy (3 mg/kg, 2-week cycle) was administered as third-line chemotherapy. Tumor markers (CEA and CA19-9) decreased after 4 cycles of nivolumab, and CT showed shrinkage of abdominal metastatic lymph node lesions after 7 cycles. However, the bilateral lower leg edema appeared at the 4th cycle of nivolumab in December, and right shoulder pain appeared and persisted from late February 2018.

**Figure 1:**
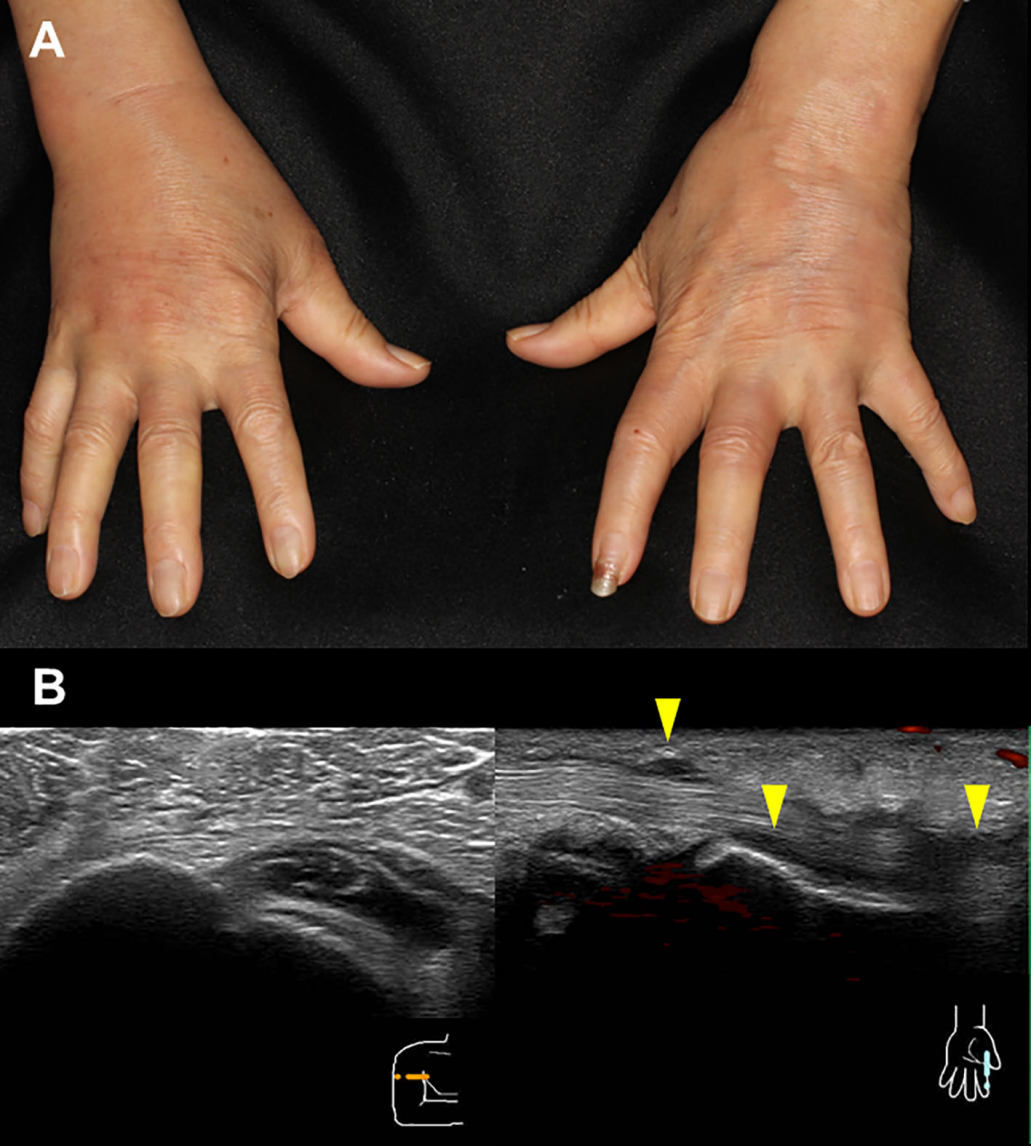
Edema of both fingers and the dorsa of both hands and erythema on the dorsal sides of the fingers **(A)**. On ultrasonographic examination, fluid retention around the biceps, synovitis of the biceps, and synovitis of the joint synovium and tendon sheath synovium are observed **(B)**.

**Figure 2:**
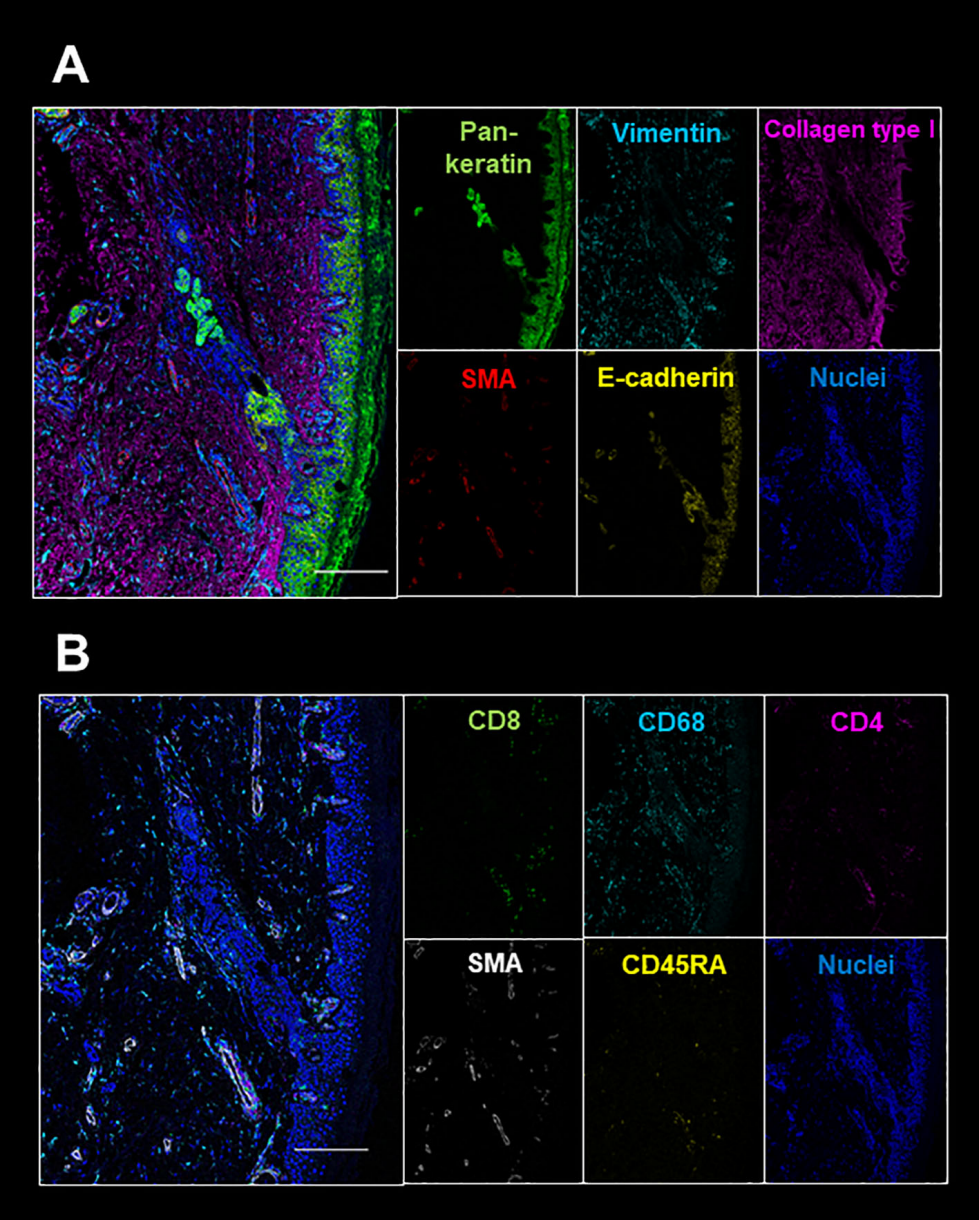
Epithelial marker (pan-keratin, E-cadherin), mesenchymal marker (vimentin, SMA), collagen type I **(A)**, and immune cell markers (CD4, CD8, CD45RA, CD68) **(B)** are shown. The white bar indicates 200 μm.

During the administration of nivolumab, edema of both fingers and the dorsa of both hands appeared in April 2018 (**Figure 1**), and pitting edema of both lower legs deteriorated. Erythema also appeared on the dorsal sides of the fingers. On ultrasonographic examination, fluid retention around the biceps, synovitis of the biceps, and synovitis of the joint synovium and tendon sheath synovium were observed (**Figure 1**). There were no abnormalities in thyroid hormone levels or the blood coagulation system. C-reactive protein was as high as 6.75 mg/dL, and rheumatoid factor was negative (8 U/mL). Serum autoantibodies including anti-nuclear antibody, anti-ds-DNA antibody, anti-SS-A antibody, anti-SS-B antibody, anti-Scl-70 antibody, anti-RNP antibody, anti-centromere antibody, anti-RNA polymerize III antibody, anti-neutrophil cytoplasmic antibody (MPO-ANCA and PR3-ANCA), anti-MDA antibody, anti-Mi-2 antibody, anti-TIF1-gamma antibody, and anti-ARS antibody were all negative. Human leukocyte antigen (HLA) typing showed HLA-A2 and HLA-CW7 serotypes. Immune cell subsets of peripheral blood mononuclear cells were analyzed using flow cytometry, as previously described (17), at three time points: prior to administration of nivolumab; after one cycle of treatment; and at the times of developing RS3PE syndrome. Activated memory, effector, and helper T cells tended to increase after administration of nivolumab. In addition, coinhibitory molecule (TIM3, TIGIT, and LAG3) and costimulatory molecule (OX40)-positive T cells also increased (**Table 1**). A skin biopsy from the dorsum of the hand showed lymphocytic infiltration around blood vessels in the surface layer of the skin and in the interstitium of the skin, and no malignant cells were observed. Multiplex immunostaining of skin tissue using imaging mass cytometry (Hyperion Imaging System, Fluidigm, South San Francisco, CA, USA) showed CD4+ or CD8+ T cells infiltrating the perivascular area and macrophages infiltrating the interstitium of the skin (**Figure 2**).

**Table 1:** Immune cell subsets of peripheral blood mononuclear cells during therapy.

(%)	Before administration of nivolumab	After administration of nivolumab	RS3PE
T cell/PBMC	50.2	58.6	58.3
CD4+T cell/T cell	71.5	71.6	87.7
naïve CD4+T cell	26.0	28.5	42.1
activated	3.8	4.6	2.8
central memory CD4+T cell	43.0	38.1	39.9
activated	5.4	8.8	9.1
effector memory CD4+T cell	29.4	32.7	17.5
activated	18.1	22.2	35.1
effector CD4+T cell	1.8	0.9	0.5
activated	29.6	31.2	23.1
Th1/CD4+T cell	18.4	15.5	12.1
activated	10.0	11.1	4.9
Th2/CD4+T cell	58.1	62.5	70.3
activated	4.7	6.7	2.3
Th17/CD4+T cell	12.0	12.3	12.9
activated	16.3	9.8	8.9
Th1/17/CD4+T cell	11.4	9.8	4.7
activated	9.3	12.4	4.0
TIM3+CD4+T cell	17.6	15.1	25.2
LAG3+CD4+T cell	4.5	5.8	21.5
TIGIT+CD4+T cell	15.0	17.3	19.9
OX40+CD4+T cell	46.6	77.1	84.1
CD8+T cell/T cell	22.9	24.5	10.6
naïve CD8+T cell	2.8	3.7	9.9
activated	25.0	19.7	13.3
central memory CD8+T cell	24.5	17.7	23.4
activated	10.4	19.4	15.5
effector memory CD8+T cell	62.2	69.0	56.8
activated	29.5	49.9	51.7
effector CD8+T cell	11.6	9.6	9.9
activated	31.6	42.6	53.3
TIM3+CD8+T cell	15.2	17.7	31.0
LAG3+CD8+T cell	2.3	9.9	16.0
TIGIT+CD8+T cell	54.5	62.7	39.8
OX40+CD8+T cell	34.3	59.9	70.4

PBMC, peripheral blood mononuclear cell; Th1, helper T1 cell; Th2, helper T2 cell; Th17, helper T17 cell; Th1/17, helper Th1/17 cell.

The changes in immune cell subsets of PBMC were evaluated at 3 time points: prior to administration of nivolumab; left column, after 1 cycle of treatment; central column, and at the time of developing RS3PE; right column. The edema and joint pain were improved in one week after the administration of prednisolone.

**Figure 3:**
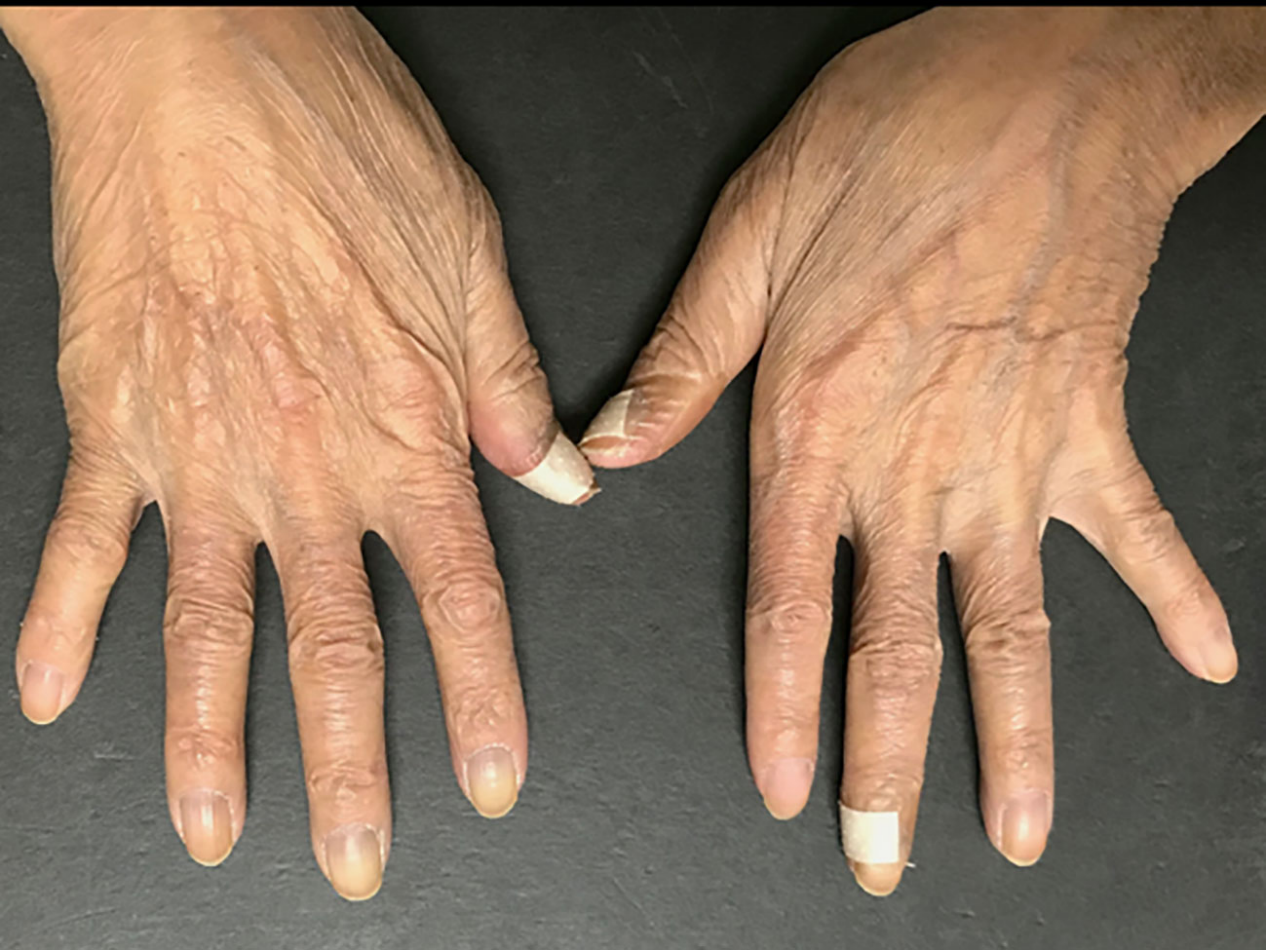
After the administration of PSL, the edema of the dorsa of the hands has improved in one week.

Based on these findings, RS3PE as an irAE of nivolumab was diagnosed. Prednisolone (PSL) 15 mg/day (0.3 mg/kg) was administered to treat the RS3PE. After the administration of PSL, the edema of the dorsa of the hands and lower legs improved in one week (**Figure 3**), and the shoulder joint pain resolved. The serum levels of matrix metalloproteinase 3, vascular endothelial growth factor (VEGF), and interleukin (IL)-6 before PSL administration were 230 ng/mL, 384 pg/mL, and 561.8 pg/mL, respectively. After the administration of PSL, they decreased to 142 ng/mL, 270 pg/mL, and 140.2 pg/mL, respectively. On ultrasonographic examination, improvement in the inflammation of the flexor tendon sheath synovium was observed. The dose of PSL was reduced to 12.5 mg/day after 2 weeks and to 10 mg/day 2 weeks later. However, after the PSL dose reduction, edema and arthralgia of both hands recurred, and the PSL dose was increased to 15 mg/day again. After the dose escalation, joint symptoms and edema improved, and administration of PSL 12.5 mg/day was continued. Nivolumab was administered for a total of 9 cycles, but the CT examination in April 2018 showed an increase in the size of the primary lesion and lymph node lesions, and PD was confirmed. Irinotecan was administered as post-treatment for AGC. After six cycles of chemotherapy, PD was confirmed by upper gastrointestinal endoscopy, but recurrence of RS3PE was not observed. The patient was transitioned to best supportive care.

## 3 Discussion

### 3.1 Diagnosis of RS3PE

RS3PE syndrome is a disease characterized by edema with bilateral synovitis. It has the following characteristics: (i) better prognosis (remitting); (ii) bilateral symmetry (symmetrical); (iii) negative for rheumatoid factor (seronegative); (iv) acute onset synovitis (synovitis); (v) pitting edema of the dorsa of the hands; (vi) onset in elderly persons; (vii) sudden onset; (viii) no bone erosion; (ix) no pain of the wrists with finger movement restriction; and (x) findings of inflammation (increased CRP or erythrocyte sedimentation rate; ESR) (10). Clinical signs of RS3PE overlap with those of PMR in terms of their response and remission to small doses of steroids. It is in fact difficult to distinguish between PMR and RS3PE because there is no specific test for RS3PE, but RS3PE is characterized by the pitting edema of the dorsa of both hands, and the present patient was diagnosed with RS3PE syndrome.

### 3.2 Pathology of RS3PE

The pathology of RS3PE is not well elucidated, but it is thought that vascular permeability due to VEGF is associated with the development of edema. It has been reported that the VEGF level in the peripheral blood of RS3PE cases was significantly higher than that of rheumatoid arthritis cases or healthy subjects, and the value was significantly higher than that of healthy subjects, and the VEGF level decreased with steroids (18). In addition, levels of the serum inflammatory cytokine IL-1, IL-6, and TNF-α were elevated, and HLA-B7, HLA-A2, HLA-CW7, and HLA-DQW2 were detected in RS3PE cases. Activated CD4+T cells (CD3+CD4+HLA-DR+), helper T1 cells (Th1; CD4+IFN-gamma+IL-4-), and type 1 cytotoxic cells (Tc1; CD8 + IFN-γ + IL-4-) were also detected in the peripheral blood of RS3PE cases (19, 20), suggesting that this disease is associated with autoimmunity. In the present case, the proportion of activated CD4+T cells also increased after administration of nivolumab. Infiltration of macrophages and T lymphocytes is observed in the synovitis of PMR, and it is considered that release of inflammatory cytokines from these cells establishes the pathological condition (21), but the pathology of the synovitis of RS3PE has not been well elucidated. IL-6, MMP3, and VEGF are associated with RS3PE synovitis, and, therefore, their serum concentrations may be useful for evaluating disease activity (18, 22). Serum levels of IL-6, MMP3, and VEGF were also high in the present case. In addition, the serum MMP3 and IL-6 concentrations decreased with steroid administration, indicating the activity of RS3PE. On the other hand, the inflammatory cytokines TNFα and IL-1beta have been reported to be less associated with RS3PE (11), and elevation of these cytokines was not observed in the present case.

### 3.3 Mechanism of the development of RS3PE

The mechanism of the development of RS3PE remains unknown. However, it has been suggested that a paraneoplastic syndrome or infection is associated with its development (23). It has been reported that parvovirus and *Mycoplasma pneumoniae* infection are triggers of RS3PE (24, 25). In the present case, there was no finding suggestive of the onset of these infections. On the other hand, the present case had AGC, and it is possible that RS3PE developed as a paraneoplastic syndrome. A paraneoplastic syndrome is thought to develop when an immune response to the tumor also occurs in the normal organs of the cancer-bearing host. Although the specific mechanism, such as common antigens, is unknown in RS3PE associated with gastric cancer, a similar mechanism may have existed in the background in the present case as well. RS3PE as a paraneoplastic syndrome can develop before the diagnosis of the tumor and develop relatively early in the clinical course of the cancer (26). The present case showed no symptoms of RS3PE during the course of the first- and second-line chemotherapies, and the first onset of RS3PE was after the administration of nivolumab as the third-line treatment, which was thought to be different from a paraneoplastic syndrome. That is, there was an auto-reactive T cell repertoire that caused RS3PE, and RS3PE did not develop until the second-line treatment, but the peripheral tolerance mechanism was disrupted by nivolumab administration, resulting in RS3PE. In the present case, coinhibitory and costimulatory molecule-positive T cells increased after the administration of nivolumab. It has been reported that chronic antigen stimulation of antigen-specific T cells increased the expression of coinhibitory and costimulatory molecules (17). Expression of these molecules on self-antigen reactive T cells might be enhanced after administration of nivolumab. In so-called isolated RS3PE, which is not paraneoplastic, activation of CD4+ or CD8+ T cells in peripheral blood and enhanced IFN-gamma production from these cells have been observed (20). This also suggests that administration of nivolumab to patients in the pre-clinical stage of RS3PE may induce RS3PE. In this case, pathological analysis for RS3PE as an irAE was performed for the first time. Infiltration of lymphocytes in the perivascular and interstitial layers of the skin was observed, which is consistent with the finding of isolated RS3PE and supports the possibility that the activation of T cells after administration of nivolumab caused an irAE in skin tissue.

## 4 Conclusion

This is the first case of a patient with gastric cancer who developed RS3PE after the administration of nivolumab. It was also shown that immune activation by nivolumab is associated with histological findings typical of RS3PE. This is useful for elucidating the mechanism of irAEs with anti-PD-1 treatment.

## Data availability statement

The raw data supporting the conclusions of this article will be made available by the authors, without undue reservation.

## Ethics statement

The studies involving humans were approved by ethics committee of Kyushu University Hospital. The studies were conducted in accordance with the local legislation and institutional requirements. The participants provided their written informed consent to participate in this study. Written informed consent was obtained from the individuals for the publication of any potentially identifiable images or data included in this article. Written informed consent was obtained from the participant for the publication of this case report.

## Author contributions

HO: Data curation, Writing – original draft, Writing – review & editing, Conceptualization, Investigation, Methodology. MK: Writing – review & editing. MU: Writing – review & editing. HA: Writing – review & editing. MI: Writing – review & editing. KT: Writing – review & editing. MA: Writing – review & editing. HN: Writing – review & editing. KA: Writing – review & editing. EB: Conceptualization, Investigation, Writing – original draft, Writing – review & editing.
